# Changes in the Composition of Biologically Active Compounds during the Ripening Period in Fruit of Different Large Cranberry (*Vaccinium macrocarpon* Aiton) Cultivars Grown in the Lithuanian Collection

**DOI:** 10.3390/plants12010202

**Published:** 2023-01-03

**Authors:** Rima Šedbarė, Izolda Pašakinskienė, Valdimaras Janulis

**Affiliations:** 1 Department of Pharmacognosy, Faculty of Pharmacy, Lithuanian University of Health Sciences, 50166 Kaunas, Lithuania; 2 Botanical Garden of Vilnius University, 10239 Vilnius, Lithuania

**Keywords:** cranberry, proanthocyanidins, flavonols, anthocyanins, triterpenoids

## Abstract

In our investigation, we evaluated the content of chlorogenic acid, proanthocyanidins, anthocyanins, flavonols, triterpenoids, and phytosterols in cranberry fruit extracts of the cultivars ‘Baifay’, ‘Early Black’, ‘Howes’, ‘Pilgrim’, ‘Red Star’, and ‘Stevens’ grown in Lithuania, as well as changes in the antioxidant activity in extracts of fruit samples of these cultivars during the period of berry maturation. The highest amount of proanthocyanidins (8.87 ± 0.57 mg EE/g EE) and flavonols (3688.52 ± 22.85 µg/g) was determined in cranberries of the cultivar ‘Howes’ harvested on 12 August. Remarkably, the highest anthocyanins content (9628.62 ± 266 µg/g) was determined in cranberries of the cultivar ‘Howes’ harvested on 22 October. The study showed that the content of phytochemical compounds in cranberries varied between 12 August and 22 October; the content of proanthocyanidins decreased by a factor of about 2, the content of chlorogenic acid decreased by a factor of about 1.3, the content of flavonols decreased by a factor of about 2, and the content of anthocyanins increased by 27 to 450 times. A strong correlation was found between the total proanthocyanidin content of cranberry fruit extracts and their in vitro antiradical and reducing activity (r = 0.781 and 0.726, respectively, *p* < 0.001). The data of our study detail the accumulation of the phytochemical composition of biologically active compounds in cranberry samples during the stages of maturity, therefore these data are significant for the assessment of harvest time of cranberry and can be applied to select cranberry cultivars for further cultivation in Lithuanian climatic conditions.

## 1. Introduction

Large cranberry (*Vaccinium macrocarpon* Aiton) belongs to the *Ericaceae* botanical family [1]. The species is widespread in eastern and central North America [2]. Cultivars of large cranberries are widely grown in industrial plantations in the USA and Canada [3]. During the recent years, human anthropogenic activities have led to a decline in the number of natural growing sites of the small cranberry (*Vaccinium oxycoccus* L.), which has resulted in the increasing popularity of the cultivation of large cranberries in the Baltic region [4,5,6]. The search for promising new cultivars of large cranberries, their selection, and their introduction for cultivation is intensifying research on the phytochemical compounds and effects of cranberries [7].

Bioactive compounds identified in cranberry fruit include proanthocyanidins, anthocyanins, phenolic acids, flavonols, and triterpene compounds [8]. Cranberries contain flavonoids, which have antibacterial [9], antioxidant [10], anti-inflammatory [11], and other biological effects. Anthocyanins determined in cranberry fruit inhibit inflammatory processes in the bowel [12], and modulate the composition of gut bacteria by reducing the abundance of *Rikenella* and *Rikenellaceae,* and increasing the abundance of *Lachnoclostridium* and *Roseburia* [13], while type A proanthocyanidins reduce the adhesion of pathogenic *Escherichia coli* bacterial strains on urinary tract urothelial cells [14]. Triterpene compounds have an anti-inflammatory effect [15] and inhibit the proliferation of cancer cells [16].

A phytochemical analysis of large cranberries samples grown in collections in Lithuania was carried out to estimate the composition of different biologically active compounds in a fragmented manner. Česonienė et al. determined the total content of anthocyanins, benzoic acid, and titratable acids in samples of large cranberry cultivars ‘Le Munyon’, ‘Searles’, ‘Pilgrim’, ‘Franklin’, ‘Early Richard’, ‘Stevens’, ‘Washington’, ‘Black Veil’, and ‘Howes’ [17]. Viškelis et al. investigated the amount of ascorbic acid and the total amounts of phenolic compounds and anthocyanins in samples of large cranberry cultivars ‘Pilgrim’, ‘Stevens’, ‘Black Veil’, and ‘Ben Lear’ collected at different stages of fruit maturation [6].

A literature search did not yield any comprehensive studies that would be performed in Lithuania on the qualitative and quantitative composition of the main groups of biologically active compounds (triterpenenoids, proanthocyanidins, flavonols, and anthocyanins) determining the pharmacological effects in cranberry fruit samples. To this end, research has been carried out and innovative instrumental analytical methodologies have been developed, optimized, and validated to analyze the qualitative and quantitative composition of cranberry fruit samples. Vilkickytė et al. proposed and validated a UPLC-DAD methodology for the analysis of anthocyanins and anthocyanidins in cranberry and other fruit samples [18]. Sedbare et al. and Urbstaite et al. proposed and validated UPLC-DAD methodologies for the analysis of flavonols, chlorogenic acid, triterpenoids, and phytosterols in large cranberry fruit samples [19,20]. The proposed methodologies for the qualitative and quantitative analysis of the composition of bioactive compounds, which are in line with modern scientific progress, have made it possible to accurately determine the phytochemical composition of fruit samples of cultivated large cranberry cultivars. The instrumental analytical tool is important for assessing the quality of medicinal plant material and the variability of bioactive compounds during the growing season, as well as for the production of quality plant material of cultivated cranberries and for the selection and introduction of cranberry cultivars.

The aim of our study was to investigate the phytochemical composition of anthocyanins, flavonols, chlorogenic acid, proanthocyanidins, triterpenoids, and phytosterols in fruit samples of large cranberry cultivars ‘Baifay’, ‘Early Black’, ‘Howes’, ‘Pilgrim’, ‘Red Star’, and ‘Stevens’ grown in Lithuania, in the collection of Vilnius University Botanical Garden, and to evaluate amount changes in these compounds during the ripening period of the fruit. 

## 2. Results and Discussion

### 2.1. Content of Anthocyanins and Anthocyanidins 

Anthocyanins have been detected in cranberry fruit samples, and have antioxidant [21,22] and anti-inflammatory effects [23]. A characteristic chromatographic profile of anthocyanins was determined in the matrix of cranberry bioactive compounds [24]. Determination of the characteristic chromatographic profile of anthocyanins can be used to determine the originality of large cranberries and to assess falsification of cranberry raw materials or preparations [25].

**Figure 1:**
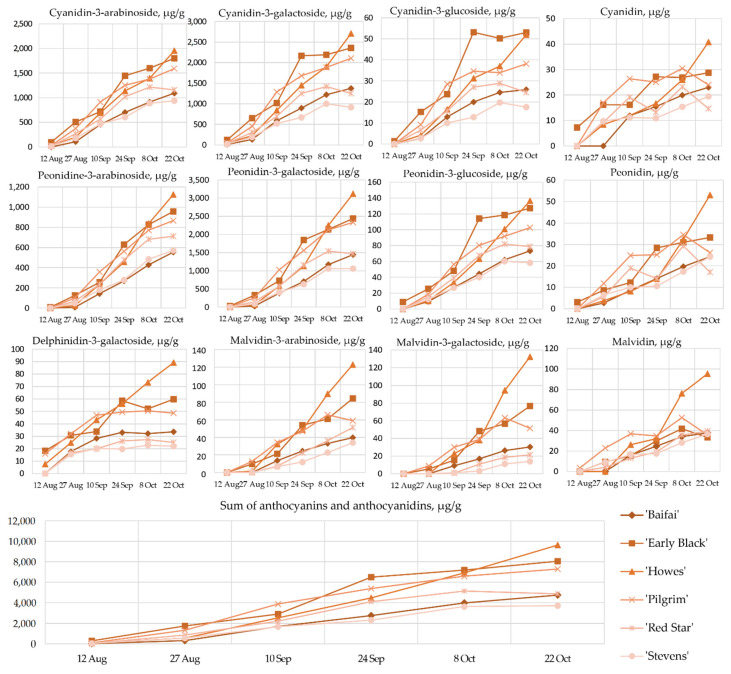
Variability of the qualitative and quantitative composition of anthocyanins and anthocyanidins in fruit samples of large cranberry cultivars during ripening.

Analysis of anthocyanins and anthocyanidins composition showed that cyanidin-3-O-galactoside, cyanidin-3-O-arabinoside, peonidin-3-O-galactoside, and peonidin-3-O-arabinoside accounted for 93.11% ± 3.4% of the sum of anthocyanins and anthocyanidins in cranberries. The composition of these four predominant glycosidic compounds of the anthocyanin group varied between August 12 and October 22: 36.89% ± 14.66% of cyanidin-3-O-galactoside, 25.84% ± 6.31% of cyanidin-3-O-arabinoside, 21.70% ± 10.78% of peonidin-3-O-galactoside, and 8.68% ± 4.46% of peonidin-3-O-arabinoside (Figure 1). The quantitative composition of the other identified compounds was lower: 2.04% ± 1.12% of delphinidin-3-galactoside, 0.55% ± 0.25% of cyanidin-3-glucoside, 1.45% ± 0.70% of peonidin-3-glucoside, 0.45% ± 0.40% of malvidin-3-galactoside, 0.57% ± 0.51% of cyanidin, 0.61% ± 0.43% of malvidin-3-arabinoside, 0.49% ± 0.28% of peonidin, and 0.72% ± 0.62% of malvidin (Figure 1). 

Viškelis et al. studied cranberry samples of the cultivars ‘Pilgrim’, ‘Stevens’, ‘Black Veil’, and ‘Ben Lear’ grown in the Lithuanian climate and determined that the content of the major anthocyanins was 33.13% ± 1.15% of peonidin-3-galactoside, 6.21% ± 1.07% of peonidin-3-arabinoside, 21.28% ± 1.05% of cyanidin-3-galactoside, and 17.57% ± 1.05% of cyanidin-3-arabinoside [6]. Česonienė et al. determined that in cranberry samples of the cultivars ‘Le Munyon’, ‘Searles’, ‘Pilgrim’, ‘Franklin’, ‘Early Richard’, ‘Stevens’, ‘Washington’, ‘Black Veil’, and ‘Howes’ grown in the Lithuanian climate, the quantitative composition of the main four anthocyanidin glycosides was 24.11% ± 2.25% of cyanidin-3-galactoside, 18.73% ± 1.32% of cyanidin-3-arabinoside, 33.29% ± 1.95% of peonidin-3-galactoside, and 16.0% ± 1.63% of peonidin-3-arabinoside [17]. These results are consistent with the anthocyanin content found in cranberries of our studied cultivars. 

Our study showed that the lowest total amounts of anthocyanin (10.45 ± 0.7 µg/g–296.49 ± 10 µg/g) were determined in cranberry fruit samples collected on 12 August. The lowest amount of total anthocyanins (10.45 ± 0.7 µg/g) was determined in the cranberry cultivar ‘Baifay’ collected on 12 August and consisted of cyanidin-3-O-galactoside. The sum of anthocyanins in cranberries of the cultivar ‘Early Black’ harvested on 12 August was 28 times higher than that determined in cranberry samples of the ‘Baifay’ cultivar harvested on the same day. In cranberries of the cultivars ‘Baifay’, ‘Early Black’, ‘Howes’, ‘Pilgrim’, ‘Red Star’, and ‘Stevens’ collected on 12 August, the levels of malvidin-3-O-galactoside, malvidin-3-O-arabinoside, and malvidin were below the limit of detection, with a more marked increase in the levels of these compounds starting on 10 September (Figure 1).

The highest total amounts of anthocyanin were determined in samples of the tested cranberry cultivars collected on 22 October. The sum of anthocyanins in fruit samples collected on 22 October ranged from 3710.58 ± 89 µg/g to 9628.62 ± 266 µg/g. The lowest amount of total anthocyanins (3710.58 ± 89 µg/g) was determined in samples of the cranberry cultivar ‘Stevens’ collected on 22 October. The highest amount of total anthocyanins (9628.62 ± 266 µg/g) was determined in cranberries of the cultivar ‘Howes’. Cranberries of the cultivar ‘Howes’ harvested on 22 October contained significantly higher levels of delphinidin-3-galactoside, malvidin-3-O-galactoside, malvidin-3-O-arabinoside, malvidin, peonidin, and cyanidin compared to the levels determined in cranberries of the other tested cranberry cultivars harvested on 22 October (Figure 1). 

**Figure 2:**
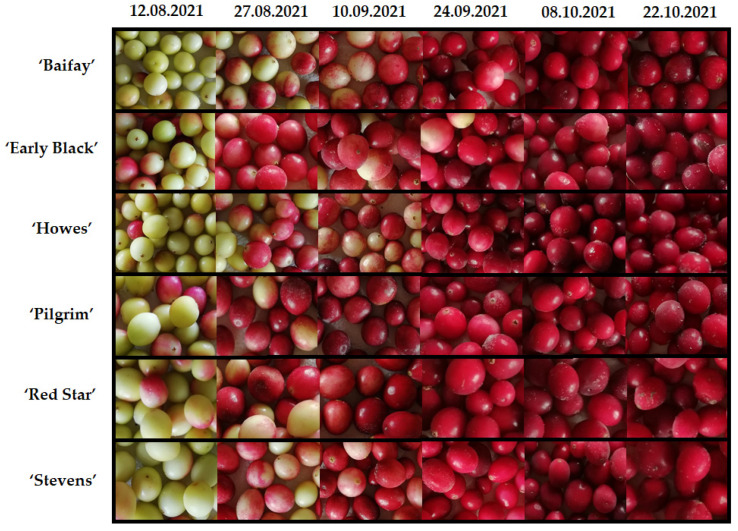
Color changes in fruit samples of large cranberry cultivars during ripening.

The content of anthocyanins and anthocyanidins in cranberries of the cultivars ‘Baifay’, ‘Early Black’, ‘Pilgrim’, ‘Howes’, ‘Red Star’, and ‘Stevens’ grown in Lithuania during the ripening period between 12 August and 22 October increased by 27 to 450 times (Figure 1). During this period, the color of the cranberries changed from greenish to bright red (Figure 2). Our results correlate with those found by other researchers [26,27]. Wang et al. investigated changes in anthocyanin and anthocyanidin content at the time of berry ripening (19 July to 17 October) in cranberries of the cultivars ‘Howes’, ‘Early Black’, ‘Stevens’, ‘Crimson Queen’, ‘Ben Lear’, ‘Demoranville’, ‘#35′, and ‘Mullica Queen’ grown in the USA under US climatic conditions [27]. The authors pointed out that the total anthocyanin amount in fruits of these cultivars increased throughout the study period and that the cultivar was an important factor influencing these changes [27]. Remarkably, the authors determined high anthocyanin levels in cranberries of the ‘Early Black’ cultivar and low anthocyanin levels in cranberries of the ‘Howes’ cultivar, which suggests environmental condition importance for anthocyanin content [27,28].

The results of our study show that the cranberry cultivars ‘Howes’, ‘Early Black’, and ‘Pilgrim’ have the highest level of anthocyanins, so these cultivars are promising for growing in Lithuanian climates as a source of anthocyanins. Furthermore, the study revealed an increasing trend in anthocyanin and anthocyanidin content, which provides knowledge on the quantitative changes in individual compounds during the ripening period and allows for selecting the optimum time for cranberry picking when the berries accumulate the highest levels of anthocyanins. Studies on the chemical composition of cranberry fruit samples allow the qualitative and quantitative composition of individual anthocyanins to be assessed and allow for the preparation of useful cranberry raw material with the highest content of anthocyanins, the biologically active compounds that determine the biological effects of cranberries.

### 2.2. Content of Flavonols

**Figure 3:**
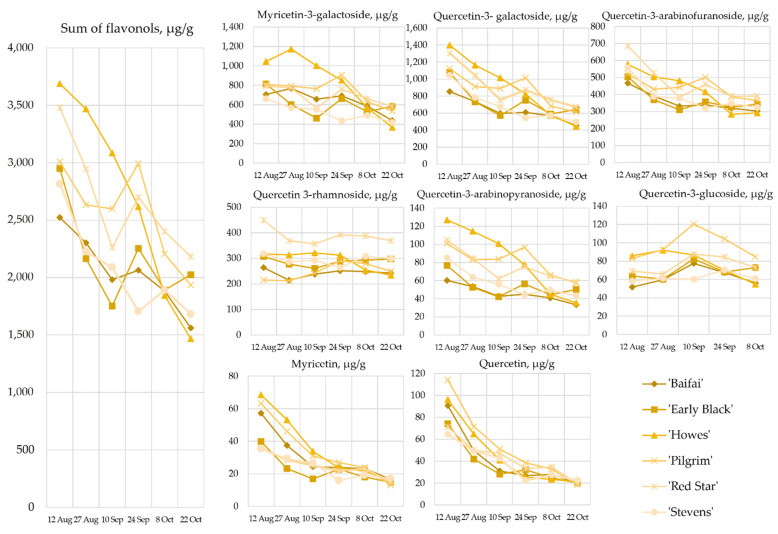
Variability of flavonols content in large cranberry samples during ripening.

Flavonols are biologically active compounds with antioxidant, cardioprotective, antibacterial, antiviral, and anticancer activities [29,30,31]. Our study showed that quercetin-3-galactoside, myricetin-3-galactoside, quercetin 3-rhamnoside, and quercetin-3-α-L-arabinofuranoside accounted for 88.69% to 92.77% of the sum of the identified flavonols in fruit samples of cranberry cultivars ‘Baifay’, ‘Early Black’, ‘Howes’, ‘Pilgrim’, ‘Red Star’, and ‘Stevens’ cultivated in Lithuania. In the studied cranberries, quercetin-3-galactoside accounted for 29.07% to 33.87% of the sum of the identified flavonols, myricetin-3-galactoside accounted for 22.73% to 33.73%, quercetin-3-α-L-arabinofuranoside—for 14.55% to 19.88%, and quercetin-3-rhamnoside—for 7.15% to 17.76% (Figure 3). The quantitative composition of the other identified compounds was lower: quercetin-3-arabinopyranoside accounted for 2.13% to 3.44% of the sum of the identified flavonols, isoquercetin—for 0.98% to 4.73%, quercetin—for 0.91% to 3.80%, and myriceting—for 0.67% to 2.27%.

Wang et. el. determined that quercetin-3-galactoside contains 31–46%, myricetin-3-galactoside contains 19–32%, quercetin-3-α-L-arabinofuranoside contains 7–17%, and quercetin-3-rhamnoside contains 7–14% of the total identified flavonols content in cranberries of different cultivars [27]. These results are consistent with the quantitative composition of flavonols identified in cranberries of our investigated cultivars.

The evaluation of flavonols showed that the total flavonol levels in cranberries ranged from 1465.56 ± 31.22 µg/g to 3688.52 ± 22.85 µg/g. The highest total flavonol content (3688.52 ± 22.85 µg/g) was determined in cranberries of the cultivar ‘Howes’ harvested on 12 August, and the lowest total flavonol content (1465.56 ± 31.22 µg/g) was determined in cranberries of the cultivar ‘Howes’ harvested on 22 October.

During the ripening period of the fruits from 12 August to 22 October, the sum of the flavonols in cranberries decreased by 1.46 to 2.52 times (Figure 3). Vvedenskaya et al. determined that during the stages of maturity from July 10 to October 11, the total flavonol content in cranberries of the cultivars ‘Stevens’ and ‘Ben Lear’ decreased by 1.4 and 1.3 times, respectively [26]. Šedbarė et al. determined that during the stages of maturity from 16 August to 15 September, the sum of the identified flavonols in cranberries of the cultivars ‘Ben Lear’, ‘Kalnciema agra’, ‘Bergman’, ‘Pilgrim’, ‘Lemunyon’, ‘Tina’, and ‘Stevens’ grown in Latvia decreased by, on average, 1.3 times [32]. 

The flavonol content in cranberries of the cultivar ‘Howes’ decreased throughout the berry picking period. Cranberry cultivars ‘Baifay’, ‘Early Black’, ‘Pilgrim’, ‘Red Star’, and ‘Stevens’ showed a different trend in total flavonol content from that seen in the ‘Howes’ cultivar. In cranberry samples of cultivars ‘Baifay’, ‘Early Black’, ‘Pilgrim’, and ‘Red Star’, an increase in total flavonols by about 1.2 times was observed on 24 September (Figure 3). In fruit samples of the cranberry cultivar ‘Stevens’, an increase in the total amount of flavonols was observed on 8 October (Figure 3).

The trends in the changes of the quantitative composition of the compounds myricetin-3-galctoside, quercetine-3-galactoside, and quercetine-3-arabinofuranoside found in the tested cranberry samples were similar to those of the total flavonol content. Between 12 August and 22 October, the amounts of myricetin and quercetin decreased by a factor of 3 and 4, respectively. Quercetin-3-rhamnoside content in fruit samples showed little variation, with a decrease of about 1.1-fold in cranberry samples of cultivars ‘Baifay’, ‘Early Black’, ‘Howes’, ‘Red Star’, and ‘Stevens’, and an increase of about 1.15-fold in cranberry samples of the cultivar ‘Pilgrim’. Wang et al. determined that quercetin-3-galactoside and myricetin-3-galactoside levels varied only slightly during the different stages of maturity in cranberries of the cultivars ‘Howes’, ‘Ben Lear’, ‘Stevens’, Early Black’, ‘Crimson Queen’, ‘Mullica Queen’, ‘Demoranville’, and ‘#35′ [27].

Studies on the phytochemical composition of cranberries allow the determination of flavonols, their quantitative composition, and trends in their content. The highest flavonol levels were found in fruit samples of the cultivars ‘Howes’, ‘Pilgrim’, and ‘Red Star’ taken at different ripening periods, indicating the importance of harvest time. The determination of the content of flavonols is important for assessing the quality of cranberry raw material and for predicting the biological effects of cranberry raw material preparations, which are determined by flavonols.

### 2.3. Quantification of Proanthocyanidins and Chlorogenic Acid

The phenolic carboxylic acids found in cranberry fruit plant material are biologically active compounds. Oszmianski et al. suggested that concentration of phenolic acids, especially of chlorogenic acid is important, as these compounds are the precursors of flavour in cranberry fruit [33]. Santana-Gálvez et al. pointed out that chlorogenic acid has biological effects such as lowering blood pressure and affecting lipid metabolism [34].

**Figure 4:**
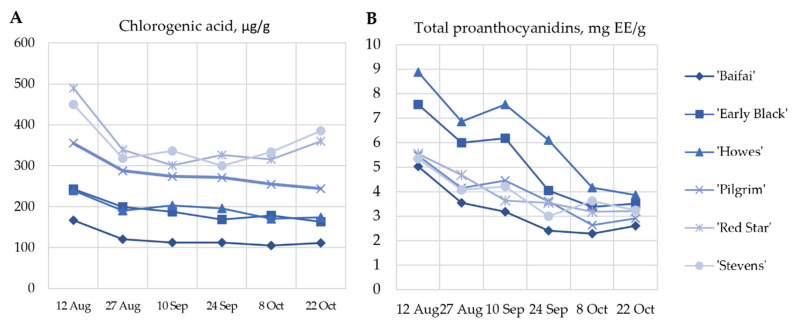
(**A**) Variability of the quantitative composition of chlorogenic acid in fruit samples of large cranberry cultivars during ripening. (**B**) Variability of the content of proanthocyanidins in large cranberry samples during ripening.

In our study, the analysis of chlorogenic acid content in cranberries showed that chlorogenic acid content varied from 104.73 ± 1 µg/g to 489.70 ± 3 µg/g (Figure 4A). The highest amount of chlorogenic acid (489.70 ± 3 µg/g) was determined in cranberries of the cultivar ‘Red Star’ harvested on 12 August. Meanwhile, the lowest quantity of chlorogenic acid (104.73 ± 1 µg/g) was determined in cranberries of the cultivar ‘Baifay’ harvested on 22 October. 

**Figure 5:**
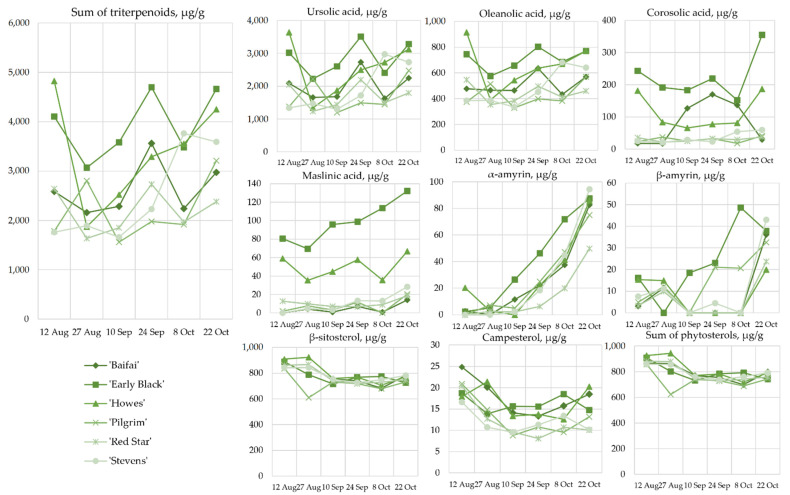
Variability of the content of triterpenoids and phytosterols in large cranberries during ripening.

During the ripening period from 12 August 2 to 27 August, the chlorogenic acid content in cranberries of cultivars ‘Baifay’, ‘Howes’, ‘Pilgrim’, ‘Early Black’, ‘Red Star’, and ‘Stevens’ decreased, on average, by a factor of 1.33 (Figure 5). During the period from 27 August to 22 October, the amount of chlorogenic acid in fruit samples of the studied cultivars slightly decreased (by 1.05-fold).

The quantity of chlorogenic acid in cranberries shows little variation during the ripening period of the cranberry fruit, thus the qualitative and quantitative evaluation of chlorogenic acid content can serve as one of the markers for the identification of the cranberry raw material and the assessment of its quality.

Cranberry fruit raw material was found to contain proanthocyanidin compounds, which are responsible for the antibacterial effect of cranberry preparations. Pérez-López et al. determined that a group of proanthocyanidins inhibit P-fimbriae synthesis and induce a bacterial deformation, on both antibiotic-susceptible and antibiotic-resistant uropathogenic *Escherichia coli* [14]. Li et al. determined that twice-daily consumption of proanthocyanidin-standardized cranberry juice may help potentiate the suppression of *Helicobacter pylori* infection [35]. In our study, the total proanthocyanidin content, estimated by spectrophotometric analysis, ranged from 2.28 ± 0.22 mg EE/g to 8.87 ± 0.57 mg EE/g in samples of cranberry fruit (Figure 4B). The highest proanthocyanidins content (8.87 ± 0.57 mg EE/g EE) was found in cranberries of the cultivar ‘Howes’ harvested on 12 August. The lowest level (2.28 ± 0.22 mg EE/g) was found in fruits of the ‘Baifay’ cultivar collected on 8 October, which was not statistically significantly different from that found in cranberries of the cultivar ‘Baifay’ harvested on 24 September.

During the cranberry ripening period from 12 August to 8 October, a decreasing trend of proanthocyanidin content was observed in cranberry samples of cultivars ‘Baifay’, ‘Howes’, ‘Pilgrim’, ‘Early Black’, ‘Red Star’, and ‘Stevens’, which showed a decrease of about a factor of 2. During the ripening period from 8 October to 22 October, the decrease in proanthocyanidin content in cranberry samples of the tested cultivars either stabilized or there was a 1.1-fold increase. Wang et al. and Vvedenskaya et al. studies confirm our results that the amount of proanthocyanidins decreased most intensively at the beginning of maturity, slightly increased at later stages of maturity [26,27]. Similar proanthocyanidins accumulation patterns were observed over fruit development in bilberry [36] and strawberry [37]. The components of proanthocyanidins biosynthesis have been studied, but the mechanism of proanthocyanidins oligomer and polymer assembly has not yet been elucidated [38]. However, there are data indicating that similar (high/low) proanthocyanidin levels in samples of cranberry cultivars are associated with genetic factors affecting flavonoid synthesis [27].

Studies on the amount of proanthocyanidins in cranberry maturity stages are relevant for the preparation of cranberry plant material. The determination of proanthocyanidin content provides an opportunity to prepare cranberries with a known proanthocyanidin content, which can be used for the production of cranberry preparations. Proanthocyanidins in cranberry fruit raw material provide antibacterial effects in the treatment and prevention of urinary tract infections, and the identification of these compounds is therefore important to produce high-quality food supplements and medicinal products of known composition and efficacy [39,40].

### 2.4. Content of Triterpenoids and Phytosterols

Triterpenoids identified in cranberry fruit are responsible for their anti-inflammatory, antitumor, and anticancer effects [41]. Chen et al. found that phytosterols lower blood cholesterol levels [42]. β-sitosterol has been found to have anti-cancer [43] and anti-inflammatory [44] effects. 

In our study, we identified and quantified triterpenoids (corosolic acid, maslinic acid, oleanolic acid, ursolic acid, β-amyrin, and α-amyrin) and phytosterols (campesterol and β-sitosterol). In cranberry fruit samples collected between August 12 and October 22, ursolic acid accounted for 75.62% ± 2.54%, oleanolic acid—for 19.50% ± 1.42%, corosolic acid—for 2.71% ± 1.95%, maslinic acid—for 0.89% ± 0.78%, α-amyrin—for 0.86% ± 0.45%, and β-amyrin—for 0.42% ± 0.32% of the total triterpenoid content. Klavins et al. confirmed that ursolic acid is the predominant triterpenoid in cranberry fruit [45].

In cranberry fruit samples, β-sitosterol content was higher (98.16% ± 1.84%) than that of campesterol (1.84% ± 0.45%). Wu et al. also indicated that β-sitosterol is the major phytosterol in cranberry fruit [46]. Šedbarė et al. found that β-sitosterol was detected in all parts of the cranberry fruit (pulp, peel, and seeds) [19]. The analysis of the quantitative composition of phytosterols showed that the content of campesterol and β-sitosterol in cranberries varied only slightly.

The changes in the total amount of triterpene compounds detected in fruit samples of the tested cranberry cultivars between 12 August and 22 October showed a mixed pattern (Figure 5). This variation was due to the content of ursolic and oleanolic acids in the samples, which accounted for the majority (95.12% ± 3.01%) of the total triterpenoid content. During the study period, the total amount of triterpenoids in cranberries ranged from 1560.43 ± 90.81 µg/g to 4827.19 ± 91.71 µg/g. The lowest amount of triterpenoids (1560.43 ± 90.81 µg/g) was determined in cranberries of the cultivar ‘Pilgrim’ harvested on September 10, which was not statistically significantly different from that found in cranberry fruits of the cultivar ‘Red Star’ harvested on 27 August. The highest triterpenoid content (4827.19 ± 91.71 µg/g) was determined in cranberries of the ‘Howes’ cultivar harvested on 12 August, which was not statistically significantly different from the levels found in fruits of the ‘Early Black’ cultivar harvested on 24 September and 22 October.

Oszmianski et al. found that the concentration of triterpenoids (oleanolic acid, betulinic acid, and ursolic acid) in fruits of cranberry cultivars ‘Stevens’ and ‘Pilgrim’ collected from 1 September to 22 September increased by a factor of 1.3 and 1.1, respectively [47]. In our study, from 28 August to 24 September, the total amount of triterpenoids in cranberry fruits of the cultivar ‘Pilgrim’ decreased by 1.4-fold, and in fruits of the cultivar ‘Stevens’, it increased by 1.2-fold. 

The changes in the amounts of the detected triterpenoids (maslinic acid, corosolic acid, β-amyrin, and α-amyrin) in cranberries varied. During the ripening period, the quantity of maslinic acid in cranberry fruits of ‘Early Black’ and ‘Howes’ cultivars was significantly higher than that in fruits of ‘Baifay’, ‘Pilgrim’, ‘Red Star’, and ‘Stevens’ cultivars (Figure 5). During the period of 12 August to 22 October, the amount of maslinic acid in cranberries of the ‘Early Black’ and ‘Howes’ cultivars increased from 80.59 ± 12.19 µg/g to 132.26 ± 10.06 µg/g and from 58.90 ± 2.16 µg/g to 66.82 ± 8.87 µg/g, respectively.

The quantity of corosolic acid in cranberries of cultivars ‘Early Black’ and ‘Howes’ was higher than that found in cranberries of cultivars ‘Pilgrim’, ‘Red Star’, and ‘Stevens’ cultivars during ripening (Figure 5). Between 12 August and 8 October, the quantity of corosolic acid in cranberries of cultivars ‘Early Black’ and ‘Howes’ decreased by a factor of 1.6 and 2.2, respectively. During the period of 8 October to 22 October, the quantity of corosolic acid in cranberries of cultivars ‘Early Black’ and ‘Howes’ increased by a factor of 2.3. The corosolic acid content in fruits of the cultivar ‘Baifay’ increased to 170.19 ± 20.2 µg/g during the period of 12 August to 24 September and decreased significantly (to 29.18 ± 2.29 µg/g) during the period of 24 September to 22 October (Figure 5).

The quantity of α-amyrin in fruits of the tested cranberry cultivars increased by up to 40-fold between 12 August and 22 October (Figure 5). The content of α-amyrin found in fruits of the tested cranberry cultivars on October 22 ranged from 49.69 ± 4.85 µg/g to 94.33 ± 2.42 µg/g. The increase in β-amyrin content in cranberries of the tested cultivars was not uniform. The highest β-amyrin content (48.64 ± 0.80 µg/g) was determined in cranberries of the cultivar ‘Early Black’ collected on 8 October.

Analysis of triterpenoids in cranberry plant material showed that the triterpenoid content in cranberry fruits varied during the ripening period. The high triterpenoids levels during the ripening of cranberry fruits were found in the samples of the ‘Early Black’ cultivar. This is possibly because the cranberries of this cultivar are smaller in size and have a larger surface area of the peel, where triterpenoids accumulate [29]. Triterpenoids occurring in fruit cuticular waxes have potential role in the protection against biotic stresses, including pathogen infections, and influencing the mechanical toughness of the fruit surface [48]. Thus, changes in the levels of triterpenoids in cranberries during ripening are greatly influenced by environmental conditions that affect triterpenoid biosynthesis [49]. 

The determination of the phytochemical compounds is important in the preparation of cranberry plant material, as it allows for the preparation of material with known levels of triterpene compounds and phytosterols, which may influence the biological effects of the preparations.

### 2.5. Antioxidant Activity

Phenolic compounds and triterpenoids found in plant matrices are natural antioxidants [50,51]. Antioxidants inhibit inflammation [52], slow the aging process, fight oxidative stress caused by free radicals [53], and reduce the risk of cancer and other chronic diseases [54]. In order to determine the changes in the antioxidant activity of fruit samples of the studied cranberry cultivars during berry ripening, it is expedient to perform in vitro assays for reducing and antiradical activity. The data obtained during these assays provide information on changes in antioxidant activity in cranberry fruit extracts during berry ripening and allow for the assessment of the linear correlation between antioxidant activity and the amount of phenolic and triterpenoid compounds detected.

In our study, the antiradical activity of 100% acetone and 70% ethanol acidified with 1% HCl extracts of cranberries was estimated by the ABTS method, and the reducing activity was tested by applying the FRAP method. The extraction of triterpenoids by aqueous mixtures of organic solvents (ethanol, acetone, and methanol) is less efficient with a lower polarity organic solvent, and therefore 100% acetone was chosen as the extraction solvent to improve the efficiency of the extraction of triterpenoid compounds from cranberry fruit samples [55,56]. Phenolic compounds are better extracted from the plant matrix using mixtures of organic solvents with water [55,57]. Klavins et al. found that the use of 70% ethanol acidified with 1% HCl for the extraction of cranberry fruit samples was effective in extracting phenolic compounds [58]. Cranberry fruit sample extracts extracted with 70% ethanol acidified with 1% HCl were selected for further analysis of proanthocyanidins, flavonols, anthocyanins, and chlorogenic acid. 

**Figure 6:**
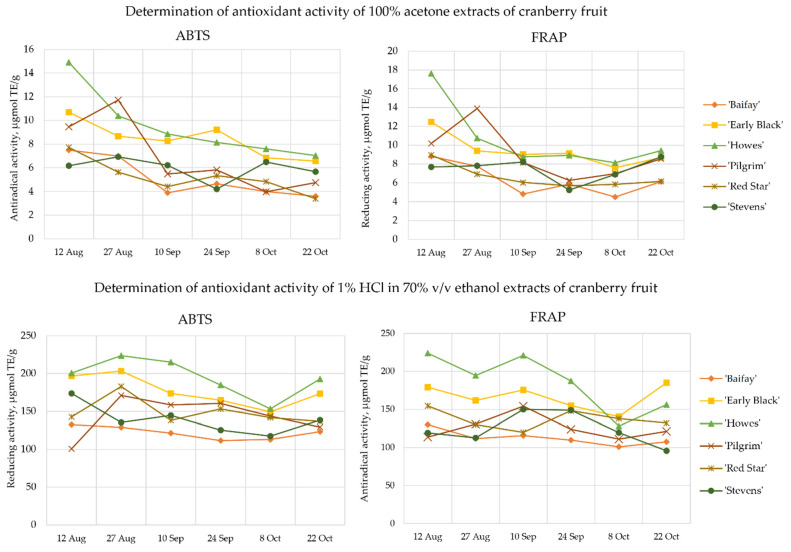
Variability of antioxidant activity in fruit samples of large cranberry cultivars during ripening.

Changes in the antioxidant activity of cranberry extracts occurring during berry ripening are shown in Figure 6. The antioxidant activity of the acetonic extracts of cranberry fruit, as assessed by ABTS and FRAP, was about 20 times lower than that of the ethanolic cranberry extracts.

The antiradical activity of cranberries acetone extracts evaluated by the ABTS method during the ripening of the berries ranged from 3.39 ± 0.38 µmol TE/g to 14.90 ± 2.10 µmol TE/g. The reducing activity of cranberries acetone extracts estimated by the FRAP method ranged from 4.51 ± 0.26 µmol TE/g to 17.61 ± 3.26 µmol TE/g. The acetone extracts of cranberry cultivar ‘Howes’ produced from cranberry samples collected on 12 August showed the strongest antiradical (14.90 ± 2.10 µmol TE/g) and reducing (17.61 ± 3.26 µmol TE/g) activity.

Acetone extracts of cranberry cultivars ‘Baifay’, ‘Early Black’, ‘Howes’, ‘Pilgrim’, and ‘Red Star’ made from fruit samples collected on 12 August showed by 2 times stronger antiradical activity and by 1.5 times stronger reducing activity than acetone extracts of cranberry fruit collected on 22 October did. Acetone extracts of fruit samples of the ‘Stevens’ cultivar showed a 1.5-fold decrease in antioxidant activity between 12 August and 24 September, and an approximately 1.5-fold increase between 24 September and 22 October.

The antiradical activity of the ethanolic cranberry extracts varied from 95.56 ± 3.74 µmol TE/g to 223.90 ± 16.11 µmol TE/g during the ripening of the berries. The extracts of the ‘Howes’ cranberry fruit samples collected on 12 August demonstrated the strongest antiradical activity (223.90 ± 16.11 µmol TE/g). The weakest antiradical activity (95.56 ± 3.74 µmol TE/g) was determined in cranberry extracts of the ‘Stevens’ cultivar made from cranberry samples collected on 22 October.

The antiradical activity of the ethanolic cranberry extracts of the cultivars ‘Early Black’ and ‘Howes’ decreased by a factor of about 1.4 between 12 August and 8 October and increased by a factor of 1.3 between 8 October and 22 October. The strongest antiradical activity of the ethanolic cranberry extracts of cultivars ‘Pilgrim’ and ‘Stevens’ was found in extracts made from cranberries harvested on 10 September. The ethanolic extracts of the cranberry cultivars ‘Baifay’ and ‘Red Star’ showed only a slight variation in antiradical activity during the study.

The reducing activity of cranberry ethanolic extracts as assessed by the FRAP method varied from 100.79 ± 13.46 µmol TE/g to 223.60 ± 14.96 µmol TE/g. The strongest reducing activity (223.60 ± 14.96 µmol TE/g) was observed for the ethanolic extracts of cranberry fruit samples of the cultivar ‘Howes’ collected on 27 August. Meanwhile, the ethanolic cranberry extracts of the cultivar ‘Pilgrim’ harvested on 12 August had the weakest antiradical activity (100.79 ± 13.46 µmol TE/g).

The reducing activity of the ethanolic cranberry extracts of the cultivars ‘Early Black’, ‘Howes’, and ‘Stevens’ decreased by a factor of about 1.3 between 12 August and 8 October and increased by a factor of about 1.2 between 8 October and 22 October. The strongest reducing activity of the ethanolic extracts of the cranberry cultivars ‘Pilgrim’ and ‘Red Star’ was found in the extracts from cranberries harvested on 27 August. The antiradical activity of the ethanolic fruit extracts of the cranberry cultivar ‘Baifay’ showed only a slight variation during the study.

We found a linear correlation between antioxidant activity and the levels of anthocyanins, flavonols, chlorogenic acid, proanthocyanidins, triterpene compounds, and phytosterols. The total amount of proanthocyanidins had a strong positive correlation with the antiradical activity determined by the ABTS method and the reducing activity determined by the FRAP method (r = 0.781, *p* < 0.001 and r = 0.726, *p* < 0.001, respectively). Proanthocyanidins identified in cranberry fruit samples contain epicatechin units in their structure, which have exceptionally strong antioxidant properties [41,59]. The strong antioxidant properties are possibly due to proanthocyanidins have more hydroxyl groups, which act as strong hydrogen donors, to receive free radicals and free radicals generated by itself form stable intramolecular hydrogen bonds with semi-quinoid free radicals and o-quinones, and thus block the chain reaction of free radicals [60]. Denev et al. found that proanthocyanidins have the strongest antioxidant activity among other phenolic compounds [61]. The strong correlation between proanthocyanidin content and antioxidant activity found in our research confirms that cranberry proanthocyanidins significantly contribute to the antioxidant properties of cranberry.

The total amount of flavonols had a moderate positive correlation with the antiradical activity determined by the ABTS method and the reducing activity estimated by the FRAP method (r = 0.476, *p* < 0.05 and r = 0.452, *p* < 0.05, respectively). We found that the total triterpenoid content had a moderate positive correlation with the antiradical activity determined by the ABTS method and the reducing activity estimated by the FRAP method (r = 0.415, *p* < 0.05 and r = 0.446, *p* < 0.05, respectively). A moderate correlation was found between the total phytosterol content in cranberry fruit extracts and the in vitro antiradical and reducing activity of their extracts (r = 0.434 and 0.447, *p* < 0.05, respectively). We found no correlation between the antioxidant activity of the extracts and the anthocyanin or chlorogenic acid content.

Oszmiański et al. investigated the linear correlation between antioxidant activity and the levels of triterpenoids, anthocyanins, flavonols, and phenolic acids found in cranberry samples of the cultivars ‘Ben Lear’, ‘Franklin’, ‘Howes’, ‘Pilgrim’, ‘Red Star’, and ‘Stevens’ [7]. The authors found that the antioxidant activity determined by the ABTS and FRAP methods had a strong positive correlation with the detected levels of triterpenoids (r = 0.852 and r = 0.736, *p* < 0.05, respectively) and flavonols (r = 0.646 and r = 0.728, *p* < 0.05, respectively) [7]. The authors reported that phenolic acid content did not correlate with the detected antiradical and antioxidant activity, but the content of anthocyanins in cranberries had a moderate positive correlation with the antioxidant activity of cranberry fruit extracts, as determined by the ABTS (r = 0.675, *p* < 0.05) and the FRAP (r = 0.614, *p* < 0.05) methods [7]. Urbštaitė et al. found that the correlation between anthocyanin quantity and antioxidant activity assessed by the FRAP method was r = 0.507 (*p* < 0.001), and by the ABTS method was r = 0.635 (*p* < 0.001) [62].

Cranberry fruit is a valuable plant material accumulating flavonoids and triterpenoids. In plant materials, it is often not possible to attribute the biological effects to a specific group of compounds or to a specific compound, as the complex of polyphenolic compounds acts synergistically [63,64]. As the chemical composition of the berry samples changes during ripening, the antioxidant activity of cranberry extracts also changes, and therefore the evaluation of the antioxidant activity in cranberry fruit samples, together with the qualitative and quantitative assessment of the phytochemical compounds of cranberries, may be important for the determination of cranberry raw material quality.

## 3. Materials and Methods

### 3.1. Raw Material

**Figure 7:**
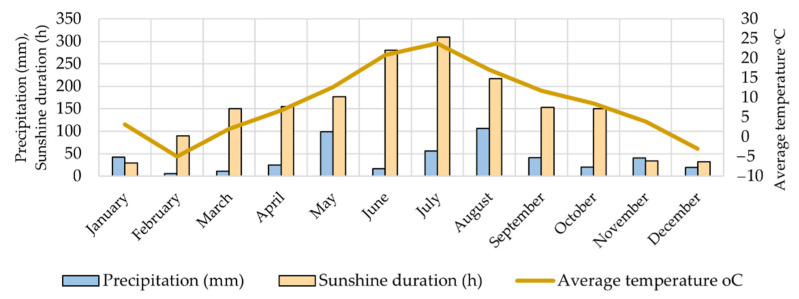
General climatic condition (temperature, precipitation, and sunshine duration) in Vilnius region during 2021.

The object of the study was large cranberry (*Vaccinium macrocarpon* Aiton) fruit samples of different cultivars grown in Lithuanian climatic conditions, in the collection of VU Botanical Garden. The variables of meteorological data (precipitation (mm), temperature (°C), and sunshine duration (h)) in the Vilnius region during 2021 are presented in Figure 7 [65].

Samples of the cultivars ‘Baifay’, ‘Early Black’, ‘Howes’, ‘Pilgrim’, ‘Red Star’, and ‘Stevens’ were harvested from the large cranberry collection field grown on Kairėnai Street 43 in Vilnius (54°43′47.3″ N 25°24′34.5″ E). Cranberry fruits were collected during berry ripening six times: on 12 August, 27 August, 10 September, 24 September, 10 October, and 22 October 2021 (Figure 2). 

**Figure 8:**
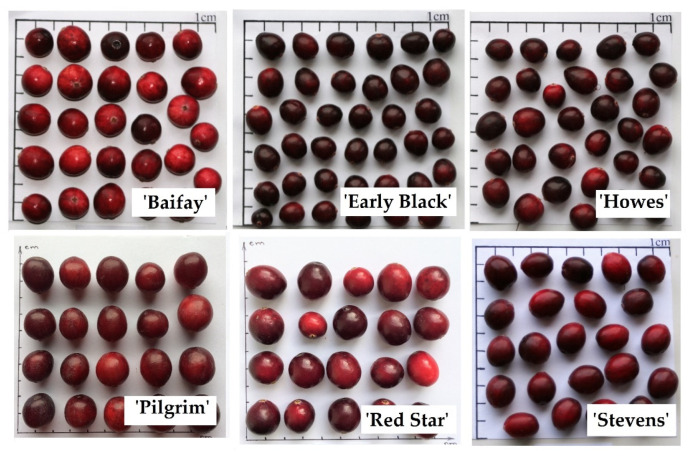
Variability of fruit sizes and shapes in large cranberry cultivars.

The fruit shapes and sizes of the cranberry cultivars ‘Baifay’, ‘Early Black’, ‘Howes’, ‘Pilgrim’, ‘Red Star’, and ‘Stevens’ are shown in Figure 8. The average weight of a fruit of the ‘Baifay’ cultivar is 1.85 ± 0.15 g of the ‘Early Black’ cultivar—1.0 ± 0.1 g, of the ‘Howes’ cultivar—1 ± 0.15 g, of the ‘Pilgrim’ cultivar—1.5 ± 0.1 g, of the ‘Red Star’ cultivar—1.35 ± 0.1 g, and of the ‘Stevens’ cultivar—1.55 ± 0.15 g.

The harvested cranberries were crushed and frozen at −20 °C in a freezer. Afterwards, the samples were transferred to a −60 °C freezer (CVF330/86, Cli-masLab SL, Barcelona, Spain). Cranberry samples were freeze-dried by Gudžinskaitė et al. described methodology [66]. Freeze-dried cranberry samples were ground into a powder in a Retsch GM 200 electric mill (Retsch GmbH, Hahn, Germany). Loss on drying was determined using the method described in the European Pharmacopoeia [67].

### 3.2. Preparation of Cranberry Extracts

Ethanol extracts were prepared for the determination of the content of flavonols, anthocyainis, chlorogenic acid and proanthocyanidins. The freeze-dried powders of each cranberry samples were extracted three times with 70% (*v/v*) ethanol containing 1% hydrochloric acid in ratio 1:20 according to Urbstaite et al. described methodology [62].

Acetone extracts were prepared for the determination of the content triterpenoids and phytosterols. The freeze-dried powders of each cranberry samples were extracted three times with 100% acetone in ratio 1:10 according to Sedbare et al. described methodology [19]. 

Prior to the chromatographic analysis, the cranberry extracts were filtered through filters with 0.22 µm pore size membrane filters (Carl Roth GmbH, Karlsruhe, Germany). 

### 3.3. Chromatographic Analysis

The analysis of the composition of anthocyanins, phenolic compounds, triterpenic compounds, and phytosterols in cranberry fruit was performed using a Waters ACQUITY Ultra High-Performance LC system (Water, Milford, MA, USA) with a photodiode array detector. The composition of anthocyanins and anthocyanidins in cranberry samples were determined using methodology described by Vilkickyte G. et al. [18]. Determination of flavonols and chlorogenic acid in cranberry samples was performed by Urbstaitė et al. described methodology [20]. Triterpenic compounds and phytosterols in cranberry samples was determined using methodology described by Šedbarė et al. [19]. 

### 3.4. Spectrophotometric Studies

#### 3.4.1. Determination of Proanthocyanidin Content

The content of proanthocyanidins was performed using the DMCA assay methodology described by the Heil et al. [68] and modified by Šedbarė ir kt. [32]. The total content of proanthocyanidins was calculated from the (–)-epicatechin (0.0625 mg/mL–1 mg/mL) calibration curve (y = 0.7021x + 0.0138; R^2^ = 0.9994) and expressed as mg/g (–)-epicatechin equivalent (EE) dry weight.

#### 3.4.2. Determination of Antioxidant Activity

An ABTS^∙+^ (radical cation decolorization study) antioxidant activity determination was performed using the methodology introduced by Re et al. [69] and described and modified by Raudone et al. [70]. A volume of 3 mL of the ABTS∙+ solution (absorbance 0.800 ± 0.02) was mixed with 5 μL and 20 μL of ethanol cranberry extract and acetone cranberry extract, respectively. The samples were kept in the dark for 30 min, then the decrease in absorbance was measured at 734 nm. The antiradical activity of the ethanolic cranberry extract was calculated from the standard curve (y = 0.00004x − 0.0823; R^2^ = 0.999) which was prepared using standard Trolox solutions of 2000–11,000 μmol/L concentration. The ABTS^∙+^ radical cation decolorization activity of the acetone cranberry extract was calculated from the standard curve (y = 0.0002x − 0.0464; R^2^ = 0.998) which was prepared using standard Trolox solutions (375–3000 μmol/L).

The FRAP (ferric reducing antioxidant power) assay was performed by Raudone et al. described methodology [71]. A volume of 3 mL of prepared FRAP reagent was mixed with 5 μL and 20 μL of ethanol cranberry extract and acetone cranberry extract, respectively. After 30 min, the absorbance was read at 593 nm using a UV-vis spectrophotometer. The reducing antioxidant power of the ethanolic cranberry extract was calculated from the standard curve (y = 0.00006x − 0.0235; R^2^ = 0.995) which was prepared from standard Trolox solutions (2000–11,000 μmol/L). The reducing activity of the acetone cranberry extract was calculated from the standard curve (y = 0.0003x − 0.0046; R^2^ = 0.993) which was prepared from standard Trolox solutions of 375–2000 μmol/L concentration. 

### 3.5. Reagents

The following reagents were used in the study: acetone, methanol (Sigma-Aldrich, Steinheim, Germany), ethanol 96% (*v/v*) (AB Stumbras, Kaunas, Lithuania), formic acid (Merck, Darmstadt, Germany), acetonitrile (Sigma-Aldrich, Steinheim, Germany), hydrochloric acid (Sigma-Aldrich, Steinheim, Germany), and 4-(Dimethylamino)cinnamaldehyde (Sigma-Aldrich, Steinheim, Germany). Reference standards for maslinic acid, corosolic acid, oleanolic acid, ursolic acid, β-Amyrin, campesterol, α-Amyrin, β-Sitosterol, chlorogenic acid, myricetin, quercetin-3-rhamnoside, quercetin-3-α-L-arabinofuranoside, and quercetin-3-α-L-arabinopyranoside were obtained from Sigma-Aldrich (Steinheim, Germany). Reference standards for delphinidin-3-galactoside, cyaniding-3-galactoside, cyaniding-3-glucoside, cyaniding-3-arabinoside, peonidin-3-galactoside, peonidin-3-arabinoside, peonidin-3-glucoside, malvidin-3-galactoside, malvidin-3-arabinoside, cyanidin chloride, peonidin chloride, malvidin chloride, and myricetin-3-galactoside were purchased from Extrasynthese (Genay, France). Reference standards for quercetin-3-galactoside and quercetin were obtained from Carl Roth (Karlsruhe, Germany). The reference standard for quercetin-3-O-glucoside was obtained from Biochemistry (Buchs, Switzerland). In addition, the following reagents were used: Trolox (6-hydroxy-2,5,7,8-tetramethylchroman-2-carboxylic acid), ABTS 2,2’-Azino-bis(3-ethylbenzothiazoline-6-sulfonic acid), sodium acetate (Scharlau Sentmenat, Spain), potassium peroxydisulfate, TPTZ (2,4,6-Tris(2-pyridyl)-s-triazine) (manufacturer: Carl Roth, (Karlsruhe, Germany), and ferric (III) chloride hexahydrate (Vaseline-Fabrik Rhenania, Bona, Gemany). The purified deionized water used in the experiments was prepared using a Milli-Q^®^ (Millipore, Bedford, MA, USA) water purification system.

### 3.6. Statistical Analysis

Data analysis was performed using computer software programs Microsoft Excel 2016 (Microsoft, Redmond, DC, USA) and SPSS Statistics 21 (IBM, Armonk, NY, USA). During the study, means and standard deviations (SD) of the three independent evaluations were calculated. To evaluate the difference in the amounts of anthocyanins, anthocyanidins, flavonols, chlorogenic acid, proanthocyanidins, and triterpenic compounds between fruit samples of different cranberry cultivars, the one-way analysis of variance ANOVA with Tukey’s test for multiple comparisons was used. Differences at *p* < 0.05 were considered significant. The correlation was determined by Pearson’s analysis. Pearson’s correlation coefficients were as follows: 0.00–0.10 was regarded as negligible correlation, 0.10–0.39—as a weak correlation, 0.40–0.69—as a moderate correlation, 0.70–0.89—as a strong correlation, and 0.90–1.00—as a very strong correlation [72].

## 4. Conclusions

The fruit samples of the large cranberry cultivars grown in the collection of Vilnius University Botanical Garden were investigated and the phytochemical composition of anthocyanins, flavonols, chlorogenic acid, proanthocyanidins, triterpenoids, and phytosterols during the ripening period of the fruit was evaluated. The timing of cranberry collection is one of the relevant factors determining the biologically active compounds quantity in the cranberry raw material. Furthermore, the different characteristics of cranberry cultivars also have a significant effect on the cranberries’ bioactive compounds composition. 

Cranberries of ‘Early Black’ and ‘Howes’ cultivars at different maturity stages contained high amounts of anthocyanins, proanthocyanidins, flavonols and triterpenoids, as well as the fruit extracts of these cultivars demonstrated high levels of antioxidant activity. Cranberry raw material prepared at the beginning of berry ripeness (in August) have higher levels of flavonols and proanthocyanidins, while cranberry raw material harvested at the end of the ripening period (in October) have high levels of anthocyanins and anthocyanidins. 

The data of the study provide new information on the phytochemical composition of biologically active compounds and patterns of their biosynthesis and accumulation in fruit samples of cranberry cultivars grown in Lithuania and allow for determining the qualitative and quantitative composition of biologically active compounds. These results can be used to provide recommendations for further introduction, selection, and cultivation of cranberries in the Lithuanian climatic conditions. Moreover, studies on the phytochemical composition of cranberry cultivars are relevant because they can lead to encourage further research into the effects of cranberry biologically active compounds for health benefits and the use of cranberry plant material in the development of new drugs.

## Data Availability

All data generated during this study are included in this article.
